# Structure Design and Kinematic Modeling of a Robotic Bird Attitude Transformation Mechanism Based on Avian Flight Characteristics

**DOI:** 10.3390/biomimetics10030131

**Published:** 2025-02-22

**Authors:** Wenyang Pu, Qiang Shen, Yiming Lu, Yaojie Yan, Yuhang Yang

**Affiliations:** School of Mechatronical Engineering, Beijing Institute of Technology, Beijing 100081, China; bit82shen@bit.edu.cn (Q.S.); 1120212037@bit.edu.cn (Y.L.); 1120211199@bit.edu.cn (Y.Y.); 1120213313@bit.edu.cn (Y.Y.)

**Keywords:** robotic bird, flight characteristics, multimodal movements, attitude transformation, kinematic model

## Abstract

Birds are capable of bidirectional changes in wing morphology, transitioning from folded to extended states or vice versa during takeoff and landing. However, most bird-like robots struggle with wing folding, resulting in poor biomimicry and an inability to meet the attitude requirements for flapping wings in multimodal movements. This paper presents a multi-motor solution with an attitude transformation mechanism based on a crank-rocker structure, enabling the wings to transition between folded and extended states while performing flapping, twisting, sweeping, bending, and their coupled motions. A kinematic model of the mechanism is developed, and the length constraints of the main linkages during key movements are derived. A prototype is designed and tested to evaluate the primary flight attitudes required for both basic and multimodal movements. The test results demonstrate that the attitude transformation mechanism, through coordinated motor operation, can replicate the wing movements of birds during different flight phases, allowing the robotic bird’s flapping wings to achieve bird-like flexibility in motion. The key angles of the wing motion were measured using a motion capture system, confirming the accuracy of the kinematic model.

## 1. Introduction

In contrast to rotary-wing and fixed-wing aircraft, robotic birds that fly by flapping offer numerous advantages, including high maneuverability, low noise, and enhanced stealth, which have attracted significant attention across various fields [1,2]. However, single-degree-of-freedom flapping suffers from issues such as poor aerodynamic performance, low efficiency, and limited endurance [3,4]. Consequently, research on robotic birds has evolved from single-degree-of-freedom flapping to multi-degree-of-freedom flight capabilities, with future developments focusing on multimodal movements.

Recent advancements in the flow physics of flapping aerodynamics have provided deeper insights into the complex interactions between wing motion and fluid dynamics. For instance, Pinapatruni et al. [5] demonstrated that an optimized tubercle design on a NACA0012 airfoil significantly improves lift and reduces drag at high angles of attack by promoting attached flow between stall cells. Similarly, Sinha et al. [6] investigated the effects of pitch angular offset on a flapping airfoil, revealing that non-zero offsets enhance cyclic time-averaged lift while reducing thrust. Furthermore, studies on the dynamics of landing maneuvers have highlighted the critical role of wing sweep and pitching motions in achieving stable perching. For example, Adhikari et al. [7] examined the effect of wing sweep on perching maneuvers, demonstrating that swept-wing configurations can significantly improve aerodynamic performance during deceleration. Additionally, Adhikari and Bhattacharya [8] investigated the dynamics of rapidly pitching plates near the ground, providing valuable insights into the fluid-structure interactions during landing. Carruthers et al. [9] further explored the mechanics and aerodynamics of perching maneuvers in large birds of prey, emphasizing the importance of wing morphing and leading-edge control surfaces in stabilizing flight during low-speed maneuvers. Their work on the use and function of leading-edge flaps in eagles [10] revealed that these structures play a crucial role in enhancing lift and reducing stall during complex maneuvers such as perching and landing. These findings underscore the importance of optimizing wing kinematics and morphology not only for steady flight but also for complex maneuvers such as landing and perching.

Bird wings exhibit four basic types of movement during flight: flapping, twisting, sweeping, and folding [11,12]. Flapping involves an up-and-down movement around an axis parallel to the flight direction. Twisting refers to rotational movement around the wing’s longitudinal axis. Sweeping describes forward and backward movement around a vertical axis perpendicular to the body, while folding involves extending and bending along the wingspan direction [13,14]. Therefore, the flapping wing motion is not simply an up-and-down movement in a single plane but a complex motion within multidimensional space. The multi-degree-of-freedom flight stage refers to the phase in which flapping wing motion transitions from planar to spatial dimensions [15,16]. During this stage, robotic birds not only enhance biomimicry but also improve aerodynamic efficiency [17,18]. Beyond basic flight capabilities, the multimodal movement stage involves additional abilities such as perching on branches, taking off and landing, and moving on the ground [19,20]. Robotic birds in this stage will closely resemble real birds both in appearance and functionality, representing the pinnacle of high biomimicry. As the critical module for generating aerodynamic forces, flapping wings directly impact the aerodynamic performance of robotic birds. Therefore, the key to designing multimodal flight robotic birds lies in the development of multi-degree-of-freedom flapping wing mechanisms [21]. However, this design process is inherently multidisciplinary, involving knowledge from mechanical engineering, structural engineering, aerodynamics, and biomimicry [22]. Consequently, exploring the design of multi-degree-of-freedom flapping wing mechanisms requires a systematic, step-by-step approach.

Zhu et al. [23] proposed three approaches to achieve three degrees of freedom in flapping wing mechanical structures: separate drive, hybrid drive, and combined drive. The separate drive approach uses different mechanisms to generate flapping, twisting, and sweeping movements. For example, Wang [24] developed a three-degree-of-freedom flapping wing mechanism utilizing a double spherical rocker hinge, where flapping and sweeping motions are realized through a reciprocating mechanism and a rack-and-pinion mechanism in series transmission, while twisting motion is transmitted via a cam mechanism. Ajanic et al. [25] employed two servo motors to independently achieve flapping and twisting motions and implemented folding motion using a four-bar linkage parallelogram mechanism.

Hybrid drive refers to the use of one mechanism to achieve any two types of movements while employing another mechanism to accomplish the third type. Zbikowski et al. [26] applied this approach to design a three-degree-of-freedom flapping wing mechanism based on a trapezoidal structure, but it suffered from transmission dead points, which affected structural stability. To mitigate these effects, Ang et al. [27] developed an improved reverse parallelogram flapping mechanism, where the two cranks of the reverse parallelogram mechanism are fixed separately on two pulleys interconnected by a crossed belt, thus avoiding dead points and enabling three degrees of freedom motion. Combined drive refers to the use of a single mechanism to simultaneously achieve all three types of movements. Zhu et al. [28] enhanced a planar four-bar linkage mechanism by designing a seven-bar eight-hinge mechanism capable of achieving three degrees of freedom coupled flapping wing motion. Additionally, Ji et al. [29] created a flapping wing mechanism that employs a spatial six-bar linkage (RRSS-SSR), enabling flapping, twisting, and sweeping motions with a single motor, and established a kinematic model for the mechanism.

The aforementioned studies have mechanized the basic flight movements of birds, enabling robotic birds to achieve multi-degree-of-freedom motion. However, they are unable to effectively simulate actions and postures involved in multimodal movements, such as transitioning the flapping wings from a folded position alongside the body to a fully extended state. To advance robotic birds into the multimodal movement stage, this paper adopts a multi-motor solution based on the crank-rocker structure. The goal is to replicate various flight stages and multimodal postures of birds. The designed attitude transformation mechanism enables the flapping wings to execute twisting, flapping, sweeping, bending, and their coupled movements, in addition to transitioning between folded and unfolded positions. This lays the foundation for robotic birds to achieve multimodal movements.

## 2. Characteristics and Analysis of Bird Flight

### 2.1. Basic Flight and Multimodal Movement

**Figure 1:**
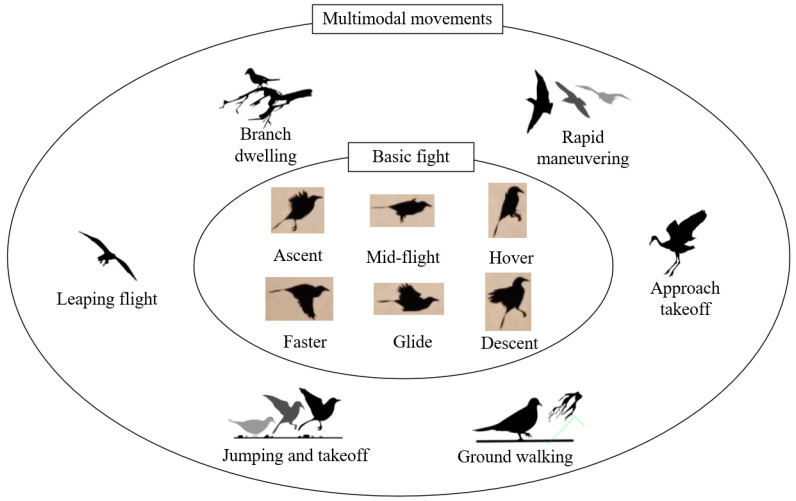
The diverse movement patterns exhibited by birds.

Apart from fundamental flight maneuvers like level flight, ascending, fast flight, hovering, leaping, and landing, birds also display multimodal movements, which enhance their ability to respond to different environmental demands. For example, to expand their habitat, they retain the ability to take off and land; to reduce energy consumption and increase flight duration, they adopt intermittent flapping-gliding flight patterns; and to evade predators, they have evolved highly maneuverable flight capabilities [20]. The multimodal movement patterns of birds are illustrated in Figure 1.

**Figure 2:**
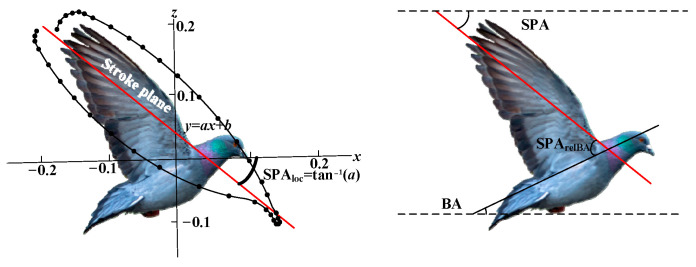
Key parameters for describing pigeon movement.

In the basic flight and multimodal motion of birds, we focus on three basic flight processes: ascent, mid-flight, and descent; and two multimodal motion processes: takeoff and landing. Taking pigeons as biomimetic models, we analyze the motion characteristics of the wings in each process based on existing research [30,31,32], laying the foundation for subsequent mechanism design. First, it is essential to define the key parameters for motion description, as shown in Figure 2.

**Figure 3:**
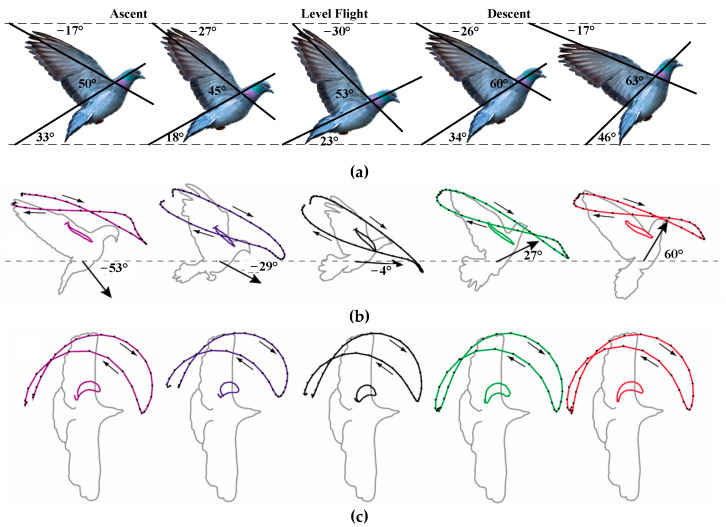
Basic flight movement diagram of pigeons. The horizontal is represented by gray dotted lines. (**a**) Displayed from top to bottom: SPA with respect to the horizontal, SPA relative to BA, and BA across all conditions. (**b**) Lateral perspectives showing the wingtip and wrist kinematics, as well as the average flight angle observed in the setup condition. (**c**) Dorsal perspective of the wingtip and wrist kinematics.

The stroke plane angle (SPA) is the angle associated with the gradient of the linear regression line calculated from the wingtip’s *x* and *z* positional coordinates relative to the shoulder. SPArelBA refers to the SPA relative to the body angle (BA). The flight postures and motion characteristics of pigeons at different stages are shown in Figure 3. Figure 3 is based on the work in reference [30].

Figure 3a shows that during steep flight (ascent and descent) and level flight in pigeons, the SPA is negative, ranging from −17° to −30°. The BA is positive, ranging from 18° to 46°, while the SPArelBA is also positive, ranging from 45° to 63°. As the steepness of flight increases, the SPA gradually decreases, while both the BA and SPArelBA increase progressively.

Figure 3b presents lateral views of the wingtip and wrist kinematics during different flight phases. It is evident that the steeper the flight, the more the wingtip path moves cranially toward the head. This suggests that as the flight angle increases, the wing extension also increases correspondingly. Additionally, as the flight angle increases, the figure-eight shape of the wingtip trajectory becomes more pronounced.

**Figure 4:**
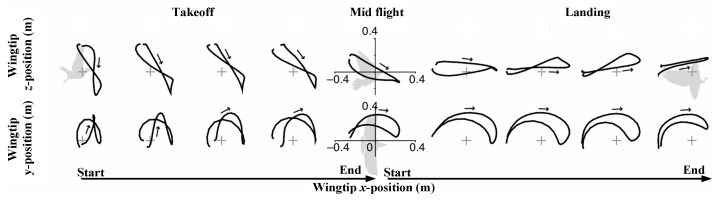
Kinematic traces for a pigeon during takeoff, mid-flight, and landing. Arrows represent the wingtip’s movement direction during the downstroke. In the top panels, the wingtip’s position relative to the shoulder is displayed for each wingbeat, with gray plus signs indicating the shoulder’s position.

Figure 3c shows the dorsal view of the wingtip and wrist kinematics. It illustrates that during ascent, descent, and level flight at different angles, the motion trajectories of the wingtip and wrist in the dorsal plane are quite similar. In all flight phases, the wingtip follows a figure-eight motion trajectory. Kinematic traces for the takeoff, mid-flight, and landing of the pigeon are shown in Figure 4.

Figure 4 shows that from takeoff to landing, the stroke Ppane (SP) rotates counter-clockwise. These traces show how the stroke planes shift from negative angles in takeoff and mid-flight to positive angles as the bird lands. During takeoff to mid-flight, the SPA is negative and gradually decreases, while during mid-flight to landing, the SPA becomes positive and gradually increases.

Throughout landing, the SP maintained a steep downward tilt during takeoff, shifting from –60 ± 5° to –47 ± 1°. This observation supports the idea that a downward-tilted SP pushes air backward, aiding forward propulsion. Similarly, during landing, the SP tilted upward, increasing from –1 ± 2° to 17 ± 7°, implying that an upward-tilted SP pushes air forward, slowing the bird. The rotations of the SP, wing planes, and tail were all linked with the rotation of the BA, suggesting that pigeons can redirect aerodynamic forces by adjusting the BA [31].

**Figure 5:**
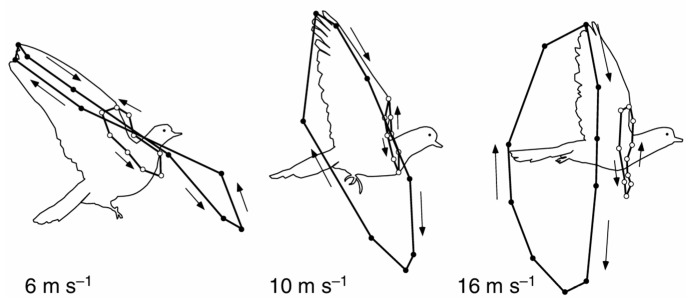
Wingtip trajectory under varying flight speeds. Arrows indicate the direction of motion.

In addition, the flight posture of pigeons is also related to flight speed. The wingtip trajectories of pigeons under varying flight speeds are presented in Figure 5 [32].

As depicted in Figure 5, with the increase in flight speed, the SPA gradually increases, the BA gradually decreases, and the wingtip trajectory transitions from a figure-eight shape to an elliptical-like shape.

### 2.2. Characteristics Analysis

The different flight phases of birds, including basic flight and multimodal movements, exhibit diverse characteristics in terms of flight parameters, wingtip path patterns, and the relationship between wingtip path and SPA. These characteristics are closely related to the aerodynamic requirements and energy distribution of each flight phase, enabling birds to achieve efficient and stable flight in various situations. Understanding these characteristics provides valuable insights into the biomechanics and flight mechanisms of birds, which is crucial for using pigeons as biomimetic models to inform the design of mechanical systems that mimic their flight capabilities.

By analyzing the motion characteristics of pigeons during different flight phases, we found that during shallow descent, level flight, or high-speed flight, the wingtip trajectory resembles an elliptical shape similar to that of the shoulder. In most other cases, the wingtip moves along a figure-eight path. Additionally, SPA decreases as the steepness of flight increases. From takeoff to landing, SP rotates counterclockwise. During the transition from takeoff to mid-flight, SPA is negative and gradually decreases, while from mid-flight to landing, SPA becomes positive and gradually increases.

To accurately simulate the wing movements of birds during different flight phases, based on the motion characteristics of birds at various flight stages, we propose that the attitude conversion mechanism should enable the flapping wings to have at least the following capabilities:

(1) The ability to perform basic flight movements, including twisting, flapping, swinging, and bending.

(2) The ability to control SPA.

(3) The ability to achieve both figure-eight and elliptical-like flapping modes.

## 3. Structural Design and Functional Analysis

### 3.1. Structural Design

**Figure 6:**
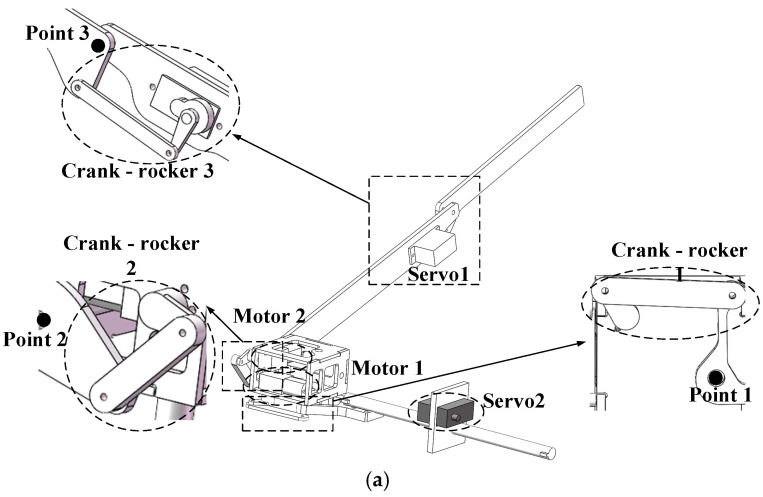

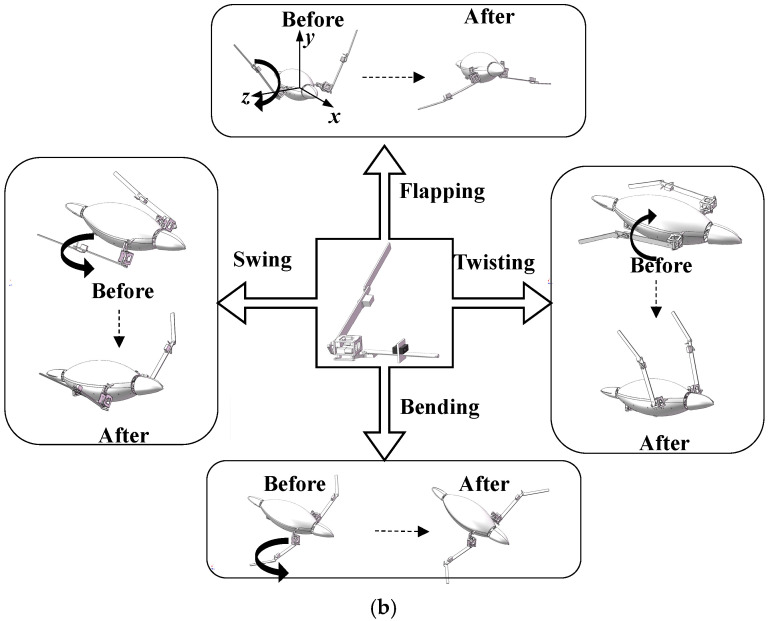
Main components of the attitude transformation mechanism (**a**) and comparison of movements before and after (**b**).

Utilizing a separate drive scheme, the attitude transformation mechanism is designed to achieve basic flight and multimodal movement capabilities similar to those of birds. This mechanism enables the wings to perform twisting, flapping, sweeping, and their coupled movements, as well as facilitates the transition of the wings from folded to unfolded or vice versa. The structure and components of the attitude transformation mechanism are shown in Figure 6.

The body coordinate system is established using the robotic bird’s center of mass as the origin and its body axis as the *x*-axis, adhering to the right-hand rule, as depicted in Figure 6b.

Twisting is exclusively regulated by Servo 2, which drives the rotation of the transmission rod, thereby inducing the connecting frame to rotate. Because the attitude transformation mechanism is rigidly fixed to the linkage, it enables rotation around the *z*-axis, which represents the twisting movement.

Flapping is realized by Motor 1 and Crank-Rocker 1. Motor 1 provides the power for flapping, with its output shaft connected to the rotating shaft. Upon activation, this connection initiates the crank’s rotation. The crank’s rotation is converted into the swing of the rocker, which causes the attitude transformation mechanism to rotate around the x-axis, enabling flapping.

Sweeping is realized by the coordinated operation of Motor 2 and Crank-Rocker 2. The output shaft of Motor 2 is connected to the sweeping shaft, driving the crank’s rotation. This motion is transmitted via the rocker. With point 2 fixed to the flapping wing, the rocker’s swing facilitates the wing’s rotation around the y-axis, enabling the sweeping motion.

The bending motion between the first and second sections of the flapping wing is controlled by Servo 1. The output shaft of the servo is connected to the folding axis. As the servo rotates, this motion is transmitted through the crank to the folding axis, which causes the rocker to swing. Since point 3 is fixed, this swing motion of the rocker allows the second section of the flapping wing to rotate around point 3, achieving folding motion.

As the flapping wing’s movements are controlled by separate motors or servos, the harmonious operation of these mechanisms enables the wing to perform flapping, twisting, sweeping, bending, and their combinations. This coordination grants the wing a level of flexibility in motion that rivals that of real birds.

### 3.2. Functional Analysis

**Figure 7:**
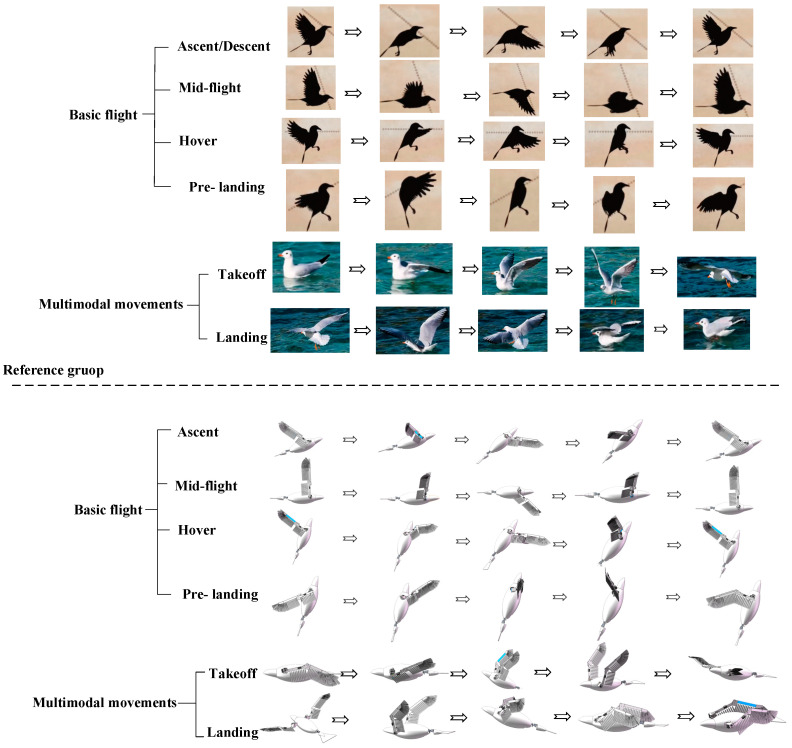
Comparison diagram of the main postures in basic flight and multimodal movement of birds with corresponding postures achieved by the attitude transformation mechanism.

Referring to the primary postures involved in basic flight and multimodal movements of birds, the corresponding postures achieved by the attitude transformation mechanism are depicted in Figure 7. The following explains the process of achieving the main postures of the attitude transformation mechanism.

Servo 1 adjusts the extension of the outer flapping wing by changing its folding angle, while Servo 2 twists the wing to the desired angle. By driving Servo 2, the SPA can be freely adjusted. Motor 1 serves as the power source for the flapping motion and operates continuously during various flight phases. The coordinated operation of Servo 2 and Motor 1 produces a coupled flapping-twisting motion, creating a figure-eight trajectory at the wingtip. Similarly, the collaboration between Motor 1 and Motor 2, which drives the coupled twisting-swinging motion, results in an elliptical-like wingtip trajectory.

During basic flight phases (ascent, descent, cruising, hovering, and pre-landing), Servo 2 first rotates to an appropriate angle, as shown in Figure 2, to achieve the desired SPA. Motor 1 is then activated to initiate the flapping motion. By adjusting the twist angle at the end of both the upstroke and downstroke in each flapping cycle, the wings achieve a figure-eight trajectory. Similarly, adjusting the swing angle at the same points produces an elliptical-like trajectory. The specific flapping trajectory can be determined based on factors such as ascent/descent angle and flight speed. Additionally, adjusting the folding angle of the flapping wings through Servo 1 modifies the relative position between the wingtip and the shoulder joint.

The takeoff phase involves the transition of the flapping wings from being folded alongside the body to fully extended, followed by ascent. Initially, the wings are positioned parallel to the sides of the body. Servo 1 adjusts the folding angle to align the first and second segments of the wings, allowing them to unfold completely. After Servo 2 twists the wings to the appropriate SPA, Motor 1 is activated, initiating the flapping motion and transitioning the wings into an ascending state for takeoff.

The landing phase involves transitioning the flapping wings from a descending state to being folded alongside the body after touchdown. The coordinated operation of the servos and motor maintains the wings in a descending state. Upon touchdown, when the upper flapping chord aligns parallel to the body’s axis, Motor 1 ceases operation, halting the flapping motion. Subsequently, Servo 2 rotates the wings clockwise to fold them alongside the body, completing the landing process.

As shown in Figure 7, the robotic bird equipped with the attitude transformation mechanism can simulate both the basic flight postures of birds and fulfill the requirements for flapping wing movement in multimodal movements.

### 3.3. Calculation of Required Torque

To address the issue of lightweight design, the body of the robotic bird is constructed from Depron foam, while the components are made from a composite material of resin and carbon fiber to balance strength and weight requirements. After considering the control system and task payload, the total estimated weight of the robotic bird is 900 g. The robotic bird primarily generates aerodynamic force through high-frequency, large-amplitude flapping. Therefore, the key factor in determining whether the flapping wing can produce the required aerodynamic force is the ability of the motor, as the power source for flapping, to provide the necessary torque.

#### 3.3.1. Design of Basic Parameters for the Flapping Wing

The basic parameters of the flapping wing, such as flapping frequency, wing span, and wing area, are estimated based on biological scaling laws derived from avian biomechanics and aerodynamics [33]. These laws describe the general relationship between body size, wing morphology, and flight performance, providing a useful framework for bio-inspired robotic design. However, it is important to note that these scaling laws are based on empirical data from a wide range of bird species and may not fully capture the variability observed in nature due to differences in flight styles, ecological niches, and evolutionary adaptations. In our study, the scaling laws were used as a starting point for the design and optimization of the flapping wing mechanism. The calculations for the wingspan, wing area, aspect ratio, and flapping frequency are shown in Equations (1)–(4), respectively.

(1) $$B=1.17m^{0.39}$$

(2) $$S=0.16m^{0.72}$$

(3) $$AR=8.56m^{0.06}$$

(4) $$f=3.87m^{-0.27}$$

where *m* represents the weight of the robotic bird, with a value of 900 g, *B* represents the wingspan, *S* is the wing area, *AR* represents the aspect ratio, and *f* is the flapping frequency. Taking into account mechanical losses, the flight efficiency of the robotic bird is approximately 25% lower than that of birds, and the flapping wing parameters are determined as shown in Table 1.

#### 3.3.2. Estimation of Flapping Wing Aerodynamic Forces

**Figure 8:**
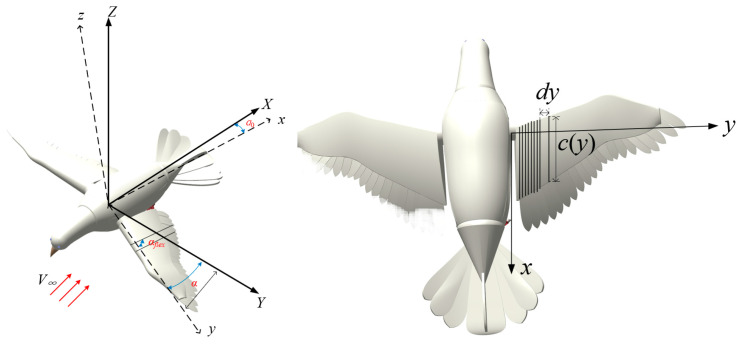
The body coordinate system and the flapping wing segmentation diagram.

To calculate the required torque for the robotic bird, it is first necessary to estimate the aerodynamic forces generated during flapping. The estimation of flapping wing aerodynamic forces was performed using quasi-steady aerodynamic laws, which provide a balance between simplicity and computational efficiency. These laws are widely used in bio-inspired robotics for their ability to approximate lift and drag forces during steady flapping motion with reasonable accuracy. Using the quasi-steady method proposed in reference [34], the aerodynamic forces are calculated, taking spanwise deformation into account. The body coordinate system and the segmentation of the flapping wing into strip elements are shown in Figure 8, where *α* represents the flapping angle, *α*_0_ is the initial installation angle, and *α_flex_* denotes the spanwise deformation angle.

First, establish the flapping motion and deformation functions as given in Equation (5) and Equation (6), respectively.

(5) $$\alpha (t)=\alpha_{\max}\cdot \pi \cdot \cos(2\cdot \pi \cdot f\cdot t)/180$$

(6) $$\alpha_{flex}(t)=\alpha_{flex\max}\cdot \pi \cdot \sin(2\cdot \pi \cdot f\cdot t)/180$$

where *α_flex_*_max_ is the maximum flapping deformation angle, *f* denotes the flapping frequency, *α*_max_ represents the maximum flapping angle, *α* is the flapping angle, and *α_flex_* represents the flapping deformation angle.

Next, establish the flapping wing contour function as shown in Equation (7).

(7) c(y)=0.154(0≤y≤0.61)

By taking the quarter-chord position of each flapping wing segment as the aerodynamic reference point, the flapping velocity and the spanwise deformation tangential velocity for each segment during flapping can be calculated as follows.

(8) $$V_{\alpha }(y,t)=y\cdot \alpha^{\prime }(t)$$

(9) $$V_{\alpha flex}(y,t)=\frac{1}{4}\cdot c(y)\cdot {\alpha^{\prime }}_{flex}(y,t)$$

Assuming the robotic bird has an initial angle of attack during flight, after the wings undergo flexible deformation, the actual flight angle of attack is

(10) $$\theta =\alpha_{0}+\alpha_{1}-\alpha_{flex}$$

where *α*_1_ is the initial angle of attack, *α*_0_ is the initial installation angle.

**Figure 9:**
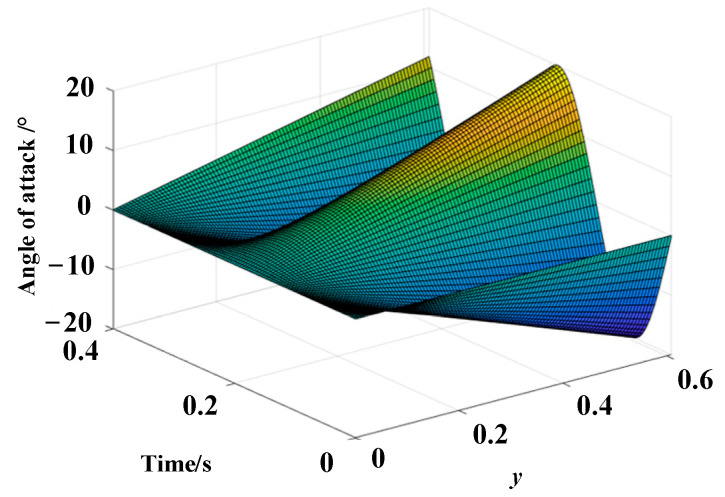
The relationship between the actual angle of attack, time, and wingspan.

Assuming an initial angle of attack of 0°, a flapping frequency of 4 Hz, a maximum flapping angle of 60°, and a maximum deformation angle of 20°, the actual flight angle *θ* as a function of spanwise coordinate *y* and time *t* can be obtained, as illustrated in Figure 9. According to the simulation results, the actual angle of attack is minimal at the wing root, equal to the sum of the initial angle of attack and the initial installation angle. As the spanwise coordinate increases, the actual angle of attack gradually increases, reaching its maximum at the wingtip.

When considering air flow velocity, the total velocity of the strip element is

(11) $$V(x,t)=\sqrt{{\left(V_{\alpha }(y,t)-V_{\alpha flex}(y,t)+V_{\infty }\sin\alpha \right)}^{2}+{\left(V_{\infty }\cos\alpha \right)}^{2}}$$

where *V*_∞_ is the air flow velocity. At this point, the actual angle of attack considering air flow velocity can be obtained as follows.

(12) $$\alpha_{c}=\arctan\left(\frac{V_{\alpha }(y,t)-V_{\alpha flex}(y,t)+V_{\infty }\sin\theta }{V_{\infty }\cos\theta }\right)$$

Based on the blade element theory, the aerodynamic lift *dF_L_* and drag *dF_D_* produced by a strip element can be calculated as follows.

(13) $$dF_{L}=\frac{1}{2}\cdot \rho \cdot C_{L}(y,t)\cdot V^{2}(y,t)\cdot c(y)dy$$

(14) $$dF_{D}=\frac{1}{2}\cdot \rho \cdot C_{D}(y,t)\cdot V^{2}(y,t)\cdot c(y)dy$$

where *ρ* represents the air density, and *C_L_* and *C_D_* denote the lift and drag coefficients, respectively, as expressed by Equations (15) and (16).

(15) $$C_{L}(y,t)=1.58\cdot \sin(2.13\cdot \alpha_{c}(y,t)-7.2)+0.225$$

(16) $$C_{D}(y,t)=-1.55\cdot \cos(2.04\cdot \alpha_{c}(y,t)-9.82)+1.920$$

We project the lift and drag in the velocity coordinate system onto the body coordinate system as follows.

(17) $$dF_{CL}=dF_{L}(y,t)\cdot \sin(\alpha_{c}-\theta )-dF_{D}(y,t)\cdot \cos(\alpha_{c}-\theta )$$

(18) $$dF_{CD}=dF_{L}(y,t)\cdot \cos(\alpha_{c}-\theta )\cdot \cos(\alpha (t))+dF_{D}(y,t)\cdot \sin(\alpha_{c}-\theta )\cdot \cos(\alpha (t))$$

By integrating the aerodynamic forces of strip elements along the spanwise direction, the overall aerodynamic forces of the flapping wing can be obtained, as shown in Equations (19) and (20).

(19) $$F_{L}=\int_{0}^{b}dF_{CL}(y,t)dy$$

(20) $$F_{T}=\int_{0}^{b}dF_{CD}(y,t)dy$$

**Figure 10:**
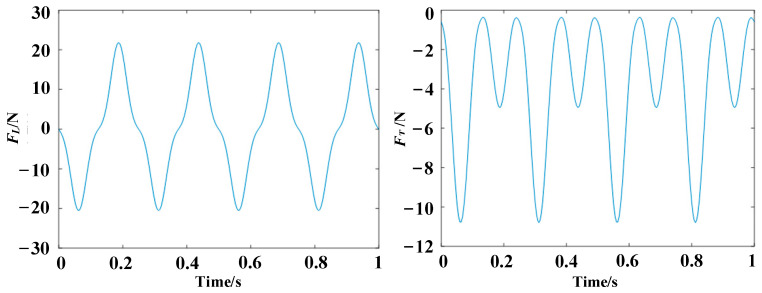
The relationship between aerodynamic forces and time.

The calculation result is depicted in Figure 10.

From Figure 10, it is determined that the maximum aerodynamic force generated by the flapping wing is approximately 22 N. The mass of a single wing is 50 g, and by taking the geometric center of the wing as the point of action, the torque required for flapping the wing is calculated as shown in Equation (21).

(21) $$N_{r}=(G+F)\cdot d$$

where *G* is the mass of the wing, *F* represents the maximum aerodynamic force, and *d* denotes the arm of force.

The calculated required torque for the flapping wing is 6.86 N∙m. Based on the requirements of torque and lightweight, the torque generated by the motor is calculated as follows.

(22) $$N_{m}=\frac{K\cdot K_{w}\cdot n}{rpm}\cdot (1-\eta )$$

where *K* is the mechanical reduction factor, with a value of 9550, *K_w_* represents the motor power; *n* is the gearbox ratio, *η* accounts for mechanical losses, and *rpm* denotes the motor’s revolutions per minute. The above describes the calculation process for determining the torque required for the robotic bird’s flapping and the torque generated by the motor, which aids in selecting appropriate motors and gearboxes. The specific parameters of main instruments are shown in Table 2.

## 4. Kinematic Modeling

### 4.1. Kinematic Modeling of Flapping Module

**Figure 11:**
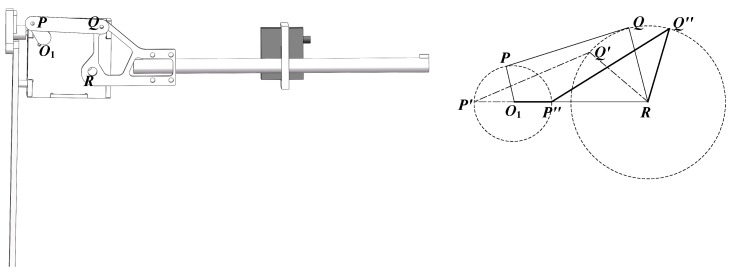
The motion parameters (**right**) and schematic diagram (**left**) of the flapping module.

The implementation details of the flapping module, including its schematic diagram and motion parameters, are presented in Figure 11.

Illustrated in Figure 11, the flapping angle depends on the flapping mechanism’s dimensions. With rotation center *O*_1_ as the origin, *O*_1_*P* denotes the crank, and *PQ* represents the rocker. The rotation of *O*_1_*P* around point *O*_1_ results in the reciprocating motion of *QR*, which defines the flapping angle.

When the crank *O*_1_*P* serves as the driving component and rotates clockwise around point *O*_1_ (0, 0) at an angular velocity of *w*_0_, traversing an angle of *w*_0_*t*, the crank-rocker mechanism will, based on mechanical principles, reach two extreme positions: *O*_1_*P’Q’R* (limit one) and *O*_1_*P*″*Q*″*R* (limit two), as depicted in Figure 11.

The angle ∠*Q’RO*_1_, which is the minimum angle, is denoted as *δ*_1_. *δ*_1_ can be expressed as Equation (24).

(23) $$\cos\delta_{1}=\frac{l_{P\prime Q\prime }^{2}-l_{P\prime R}^{2}-l_{Q\prime R}^{2}}{-2\cdot l_{P\prime R}\cdot l_{Q\prime R}}$$

(24) $$\delta_{1}=\arccos\left(\frac{l_{P^{\prime }Q}^{2}-l_{P^{\prime }R}^{2}-l_{Q^{\prime }R}^{2}}{-2\cdot l_{P\prime R}\cdot l_{Q\prime R}}\right)$$

The angle ∠*Q*″*RP*″, which is the maximum angle, is referred to as *δ*_2_. *δ*_2_ can be calculated using Equation (26).

(25) $$\cos\delta_{2}=\frac{l_{P\prime \prime Q\prime \prime }^{2}-l_{P\prime \prime R}^{2}-l_{Q\prime \prime R}^{2}}{-2\cdot l_{P\prime \prime R}\cdot l_{Q\prime \prime R}}$$

(26) $$\delta_{2}=arc\left(\frac{l_{P\prime \prime Q\prime \prime }^{2}-l_{P\prime \prime R}^{2}-l_{Q^{\prime \prime }R}^{2}}{-2\cdot l_{P\prime \prime R}\cdot l_{Q\prime \prime R}}\right)$$

The above relationship is valid only if the following condition is fulfilled.

(27) $$L_{\max}+L_{\min}<L\prime +L\prime \prime$$

where *L*_min_ is the shortest length among *O*_1_*R*, *PQ*, *OP*, and *QR*; and *L*_max_ is the longest length among *O*_1_*R*, *PQ*, *OP*, and *QR*. *L’* and *L″* refer to the lengths of the remaining links. The flapping angle can be formulated as follows.

(28) $$\delta =\delta_{2}-\delta_{1}=\arccos\left(\frac{l_{P\prime \prime Q\prime \prime }^{2}-l_{P\prime \prime R}^{2}-l_{Q\prime \prime R}^{2}}{-2\cdot l_{P\prime \prime R}\cdot l_{Q\prime \prime R}}\right)-\arccos\left(\frac{l_{P\prime Q\prime }^{2}-l_{P\prime R}^{2}-l_{Q\prime R}^{2}}{-2\cdot l_{P\prime R}\cdot l_{Q\prime R}}\right)$$

### 4.2. Kinematic Modeling of the Sweeping Module

**Figure 12:**
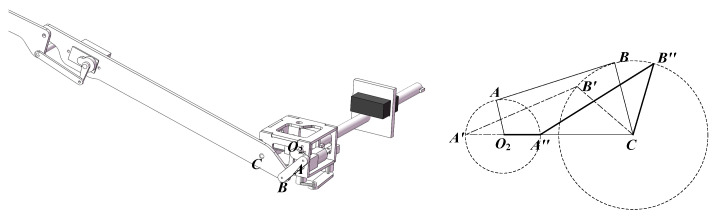
The motion parameters (**right**) and schematic diagram (**left**) of the sweeping module.

The implementation details of the sweeping module, including its schematic diagram and motion parameters, are presented in Figure 12.

Illustrated in Figure 12, the sweeping angle depends on the sweeping mechanism’s dimensions. With rotation center *O*_2_ as the origin, *O*_2_*A* denotes the crank, and *AB* represents the rocker. The rotation of *O*_2_*A* around point *O*_2_ results in the reciprocating motion of *CB*, which defines the sweeping angle.

When the crank *O*_2_*A* serves as the driving component and rotates clockwise around point *O*_2_ (0, 0) at an angular velocity of *w*_0_, traversing an angle of *w*_0_*t*, the crank-rocker mechanism will, based on mechanical principles, reach two extreme positions: *O*_2_*A’B’C* (limit one) and *O*_2_*A″B″C* (limit two), as depicted in Figure 12.

The angle ∠*B’CO*_2_, which is the minimum angle, is denoted as *θ*_1_. *θ*_1_ can be expressed as Equation (30).

(29) $$\cos\theta_{1}=\frac{l_{A\prime B\prime }^{2}-l_{A\prime C}^{2}-l_{B\prime C}^{2}}{-2\cdot l_{A\prime C}\cdot l_{B\prime C}}$$

(30) $$\theta_{1}=\arccos\left(\frac{l_{A\prime B\prime }^{2}-l_{A\prime C}^{2}-l_{B\prime C}^{2}}{-2\cdot l_{A\prime C}\cdot l_{B\prime C}}\right)$$

The angle ∠*B*″*CO*_2_, which is the maximum angle, is referred to as *θ*_2_. *θ*_2_ can be calculated using Equation (32).

(31) $$\cos\theta_{2}=\frac{l_{A\prime \prime B\prime \prime }^{2}-l_{A\prime \prime C}^{2}-l_{B\prime \prime C}^{2}}{-2\cdot l_{A\prime \prime C}\cdot l_{B\prime \prime C}}$$

(32) $$\theta_{2}=\arccos\left(\frac{l_{A\prime \prime B\prime \prime }^{2}-l_{A\prime \prime C}^{2}-l_{B\prime \prime C}^{2}}{-2\cdot l_{A\prime \prime C}\cdot l_{B\prime \prime C}}\right)$$

To ensure the validity of the above relationship, the following requirement must be met.

(33) $$L_{\max}+L_{\min}<L\prime +L\prime \prime$$

where *L*_min_ is the shortest length among *O*_2_*A*, *AB*, *BC*, and *O*_2_*C*; and *L*_max_ is the longest length among *O*_2_*A*, *AB*, *BC*, and *O*_2_*C*. *L’* and *L″* refer to the lengths of the remaining links. The sweeping angle can be calculated as in Equation (34).

(34) $$\theta =\theta_{2}-\theta_{1}$$

### 4.3. Kinematic Modeling of the Bending Module

**Figure 13:**
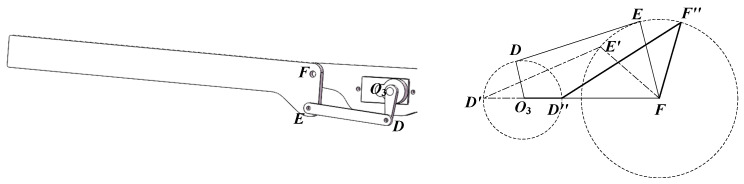
The motion parameters (**right**) and schematic diagram (**left**) of the bending module.

The implementation details of the bending module, including its schematic diagram and motion parameters, are presented in Figure 13.

Illustrated in Figure 13, the bending angle depends on the bending mechanism’s dimensions. With rotation center *O*_3_ as the origin, *O*_3_*D* denotes the crank, and *DE* represents the rocker. The rotation of *O*_3_*D* around point *O*_3_ results in the reciprocating motion of *EF*, which defines the sweeping angle.

When the crank *O*_3_*D* serves as the driving component and rotates clockwise around point *O*_3_ (0, 0) at an angular velocity of *w*_0_, traversing an angle of *w*_0_*t*, the crank-rocker mechanism will, based on mechanical principles, reach two extreme positions: *O*_3_*D’E’F* (limit one) and *O*_3_*D″E″F* (limit two), as depicted in Figure 13.

The angle ∠*E’FO*_3_, which is the minimum angle, is denoted as *ε*_1_. *ε*_1_ can be expressed as Equation (36).

(35) $$\cos\varepsilon_{1}=\frac{l_{D\prime E\prime }^{2}-l_{D\prime F}^{2}-l_{E\prime F}^{2}}{-2\cdot l_{D\prime F}\cdot l_{E\prime F}}$$

(36) $$\varepsilon_{1}=\arccos\left(\frac{l_{D\prime E\prime }^{2}-l_{D\prime F}^{2}-l_{E\prime F}^{2}}{-2\cdot l_{D\prime F}\cdot l_{E\prime F}}\right)$$

The angle ∠*F*″*FO*_3_, which is the maximum angle, is referred to as *ε*_2_. *ε*_2_ can be calculated using Equation (38).

(37) $$\cos\varepsilon_{2}=\frac{l_{D\prime \prime F\prime \prime }^{2}-l_{D\prime \prime F}^{2}-l_{F\prime \prime F}^{2}}{-2\cdot l_{D\prime \prime F}\cdot l_{F\prime \prime F}}$$

(38) $$\varepsilon_{2}=\arccos\left(\frac{l_{D\prime \prime F\prime \prime }^{2}-l_{D\prime \prime F}^{2}-l_{F\prime \prime F}^{2}}{-2\cdot l_{D\prime \prime F}\cdot l_{F\prime \prime F}}\right)$$

For the above relationship to hold, the following condition must be satisfied.

(39) $$L_{\max}+L_{\min}<L\prime +L\prime \prime$$

where *L*_min_ is the shortest length among *O*_3_*D*, *DE*, *EF*, and *O*_3_*F*; and *L*_max_ is the longest length among *O*_3_*D*, *DE*, *EF*, and *O*_3_*F*. *L’* and *L″* refer to the lengths of the remaining links. The bending angle can be expressed as in Equation (40).

(40) $$\varepsilon =\varepsilon_{2}-\varepsilon_{1}$$

## 5. Verification of Kinematic Model Accuracy and Performance Experiment of Prototype

### 5.1. Kinematic Model Accuracy Experiment

Based on target angles of 50° for bending, 105° for sweeping, and 120° for flapping, the main component dimensions were calculated using the kinematic model, as presented in Table 3.

**Figure 14:**
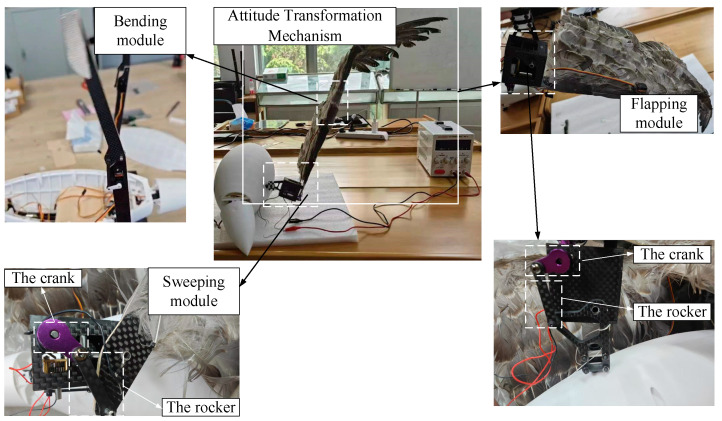
Diagram of the attitude transformation mechanism and its main functional modules.

Following the aforementioned design method and additive manufacturing, a prototype of the attitude transformation mechanism has been produced. The prototype is made from carbon fiber-reinforced nylon composite materials. Feathers, arranged in a style resembling those of avian species like eagles, are fashioned from duck feathers. The overall configuration and main functional modules of the attitude transformation mechanism are depicted in Figure 14.

**Figure 15:**
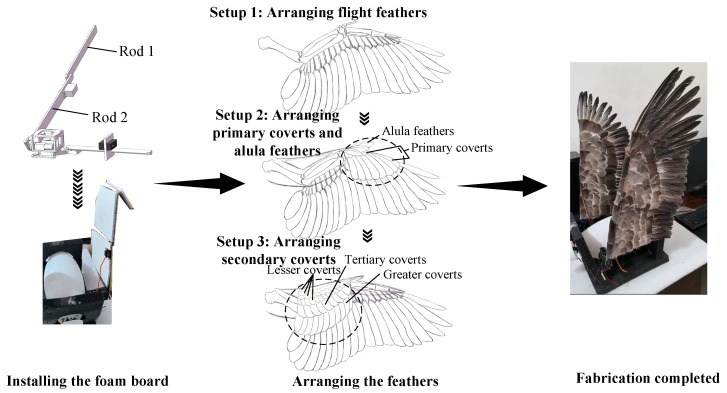
Flapping wing design methodology and process.

The flapping wing design methodology and process, as shown in Figure 15, begins by installing foam boards on the main rods 1 and 2 of the flapping wing module. Then, based on the arrangement of bird feathers, feathers (duck feathers) are arranged on the foam boards. The result is a bio-inspired flapping wing.

**Figure 16:**
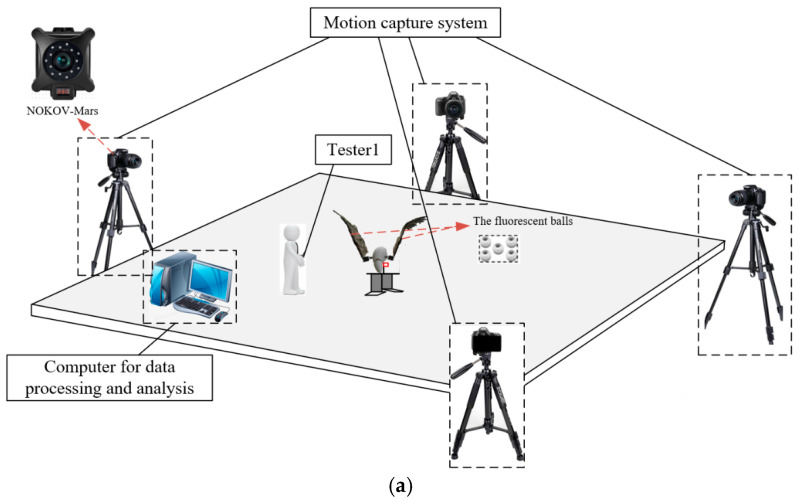

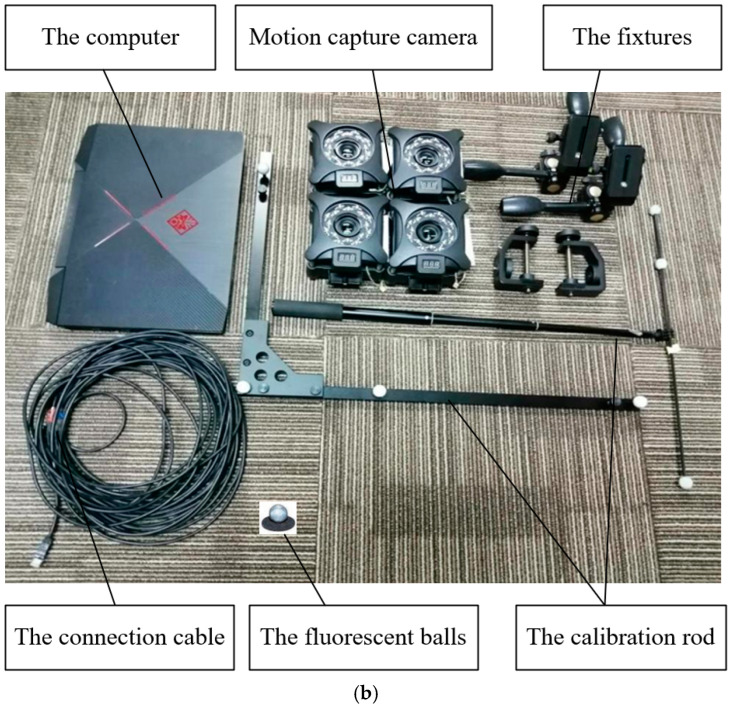
The test scenario (**a**) and primary testing equipment (**b**).

To measure motion parameters of the attitude transformation mechanism, the prototype is positioned within a 5 m × 5 m × 15 m test space. Four infrared cameras (NOKOV-Mars) is installed around the prototype. The measurement data are transmitted to the computer through a wireless network. The main testing equipment is shown in Figure 16.

**Figure 17:**
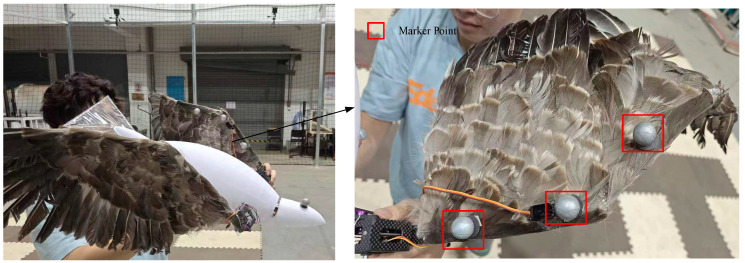
Schematic diagram of installation position of fluorescent ball.

To monitor important spatial points during the operation of the attitude transformation mechanism, fluorescent balls are installed at key positions. Following the measured motion parameters, fluorescent balls are placed along the wing’s leading edge. One is located at the center of the wing, another is at the junction between the first and second wing sections, and the last one is at the center of the second wing section, as depicted in Figure 17. To minimize the potential errors caused by scattering in the use of fluorescent balls for position tracking, several measures were implemented. First, the fluorescent balls were coated with a high-quality retroreflective material to maximize light return to the cameras and reduce diffuse reflection. Second, the NOKOV-Mars motion capture system was equipped with infrared cameras and optical filters specifically targeting the wavelength of the reflected light, thereby reducing interference from ambient light and scattering. Third, the experimental environment was carefully controlled to minimize ambient light and reflective surfaces. Finally, the system was calibrated using a calibration wand with known dimensions, and an error correction model was applied during data post-processing to compensate for any residual scattering effects. These measures ensured that the positional tracking accuracy remained within an acceptable range, with a maximum error of less than 1 mm. The motion parameters are measured by means of the Xingying 1.4.0.x software. This measurement system offers a distance accuracy of 1 mm and an angular measurement error of ±1°.

The NOKOV-Mars motion capture system was calibrated prior to the experiments to ensure accurate spatial measurements. A calibration wand with known dimensions was used to establish the coordinate system and align the cameras. Static calibration was performed using a calibration frame, followed by dynamic calibration with the calibration wand to optimize camera parameters and minimize spatial errors (typically <0.1 mm). All cameras were synchronized via a master controller, which sent synchronization signals to ensure simultaneous data capture. Data from the cameras were transmitted to the NOKOV-Mars software platform XING 1.4.0.4612, where they were aligned using timestamps to achieve a maximum delay of 10 milliseconds. This setup ensures that the motion of the fluorescent balls is accurately replicated in the software, enabling reliable tracking of the wing’s motion parameters.

**Figure 18:**
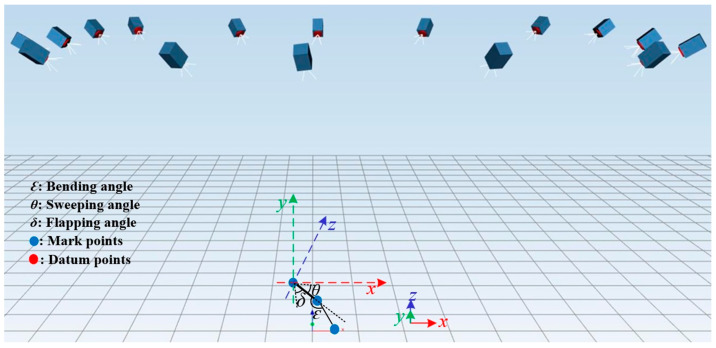
Schematic diagram of key motion parameters.

Experiments were conducted to measure the sweeping angle, flapping angle, and bending angle, with each angle measured three times to ensure accuracy. Then, we calculate the average of the three measurements for each specific angle, and this average value will be regarded as the final experimental result for that angle. The schematic illustration of the key motion parameters is presented in Figure 18.

The experimental results, calculated theoretical values, and comparisons are shown in Table 4.

Table 4 reveals that the theoretical flapping angle is 120°, differing by 3.4% from the experimental value of 116°. The theoretical bending angle is 50°, deviating by 3.8% from the experimental value. The theoretical sweeping angle is 105°, with a deviation of 2.7% from the experimental value. As a result, the maximum experimental error was 3.8%, reflecting a high degree of measurement accuracy. This error may be associated with the manufacturing precision of the main components. The experimental results support the accuracy of the kinematic model in this paper.

### 5.2. Wing Tracking Experiments

The NOKOV-Mars motion capture can record data at a frequency of 300 Hz. It is equipped with four cameras, which are capable of tracking a 28 by 28 m capture volume. The data measured by the cameras are then transmitted to the Xingying 1.4.0.x software, which replicates the motion with a 10 millisecond delay.

In order to observe the wingtip trajectory more clearly, we exported all the recorded point locations during one flapping cycle of the ornithopter and used MATLAB R2022a to perform normalization processing to enhance the clarity of the wingtip trajectory. As explained in Section 2, alterations in the wingtip path direction and SPA can reveal the mechanisms by which a flapping wing generates lift and thrust. Unlike most ornithopters, which can only achieve flapping with a single degree of freedom, the attitude conversion device we designed has four degrees of freedom of motion. This allows it to simulate the wingtip motion trajectory more accurately under different SPAs and motion functions.

**Figure 19:**
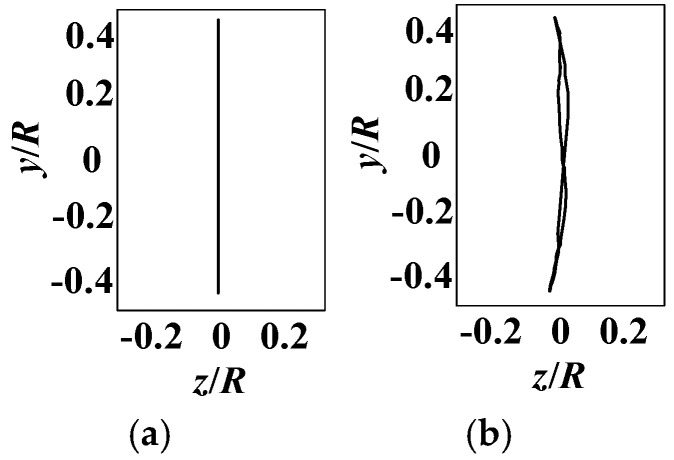
Comparison of vertical fluttering wing tip trajectory between rigid (**a**) and flexible (**b**) materials. The rigid material is carbon fiber resin composite, and the flexible material is duck feathers.

First, the motion trajectory of the wing rod without feathers was measured. The wing rod, made of a carbon fiber resin composite material, has a Young’s modulus of approximately 200 GPa, providing high bending stiffness. During the flapping motion, it undergoes almost no deformation and can be considered a rigid material. When the wing performs vertical flapping without twisting, the test results, as shown in Figure 19a, indicate that the motion trajectory is linear, with the trajectory length positively correlated with the flapping angle.

Next, feathers were attached to the wing rod, and the wingtip trajectory was measured under the same motion function. The results, shown in Figure 19b, reveal that the feathers, with a maximum Young’s modulus of approximately 3 GPa [35], can be considered a flexible material compared to the wing rod. The tests demonstrated that the wingtip trajectory is no longer linear but forms a figure-eight curve. This is likely due to the passive deformation of the flexible material under aerodynamic forces.

**Figure 20:**
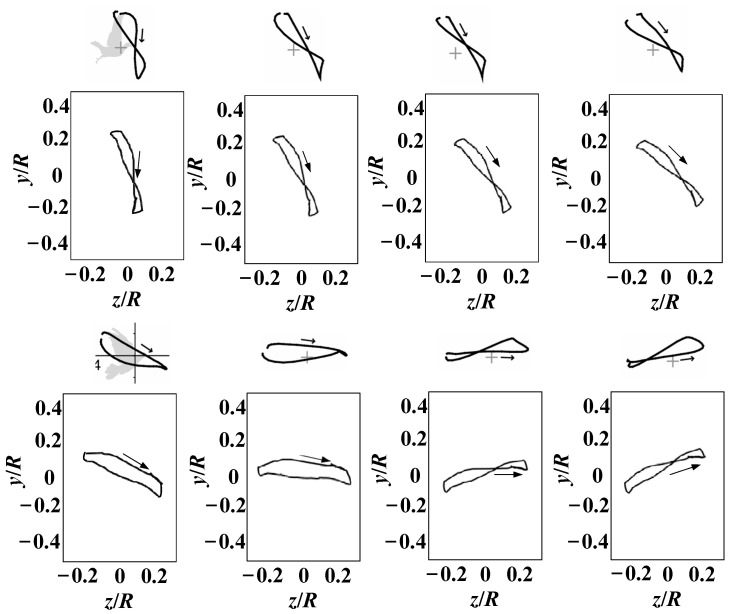
Normalized tip paths of flapping wings in the *zy* plane and comparison with the actual wingtip trajectory of pigeons, *R* = 0.614 m.

After identifying the motion trajectory of the flexible wingtip, we introduced twisting and swinging motions in addition to flapping. By adjusting the SPA through twisting and utilizing the coupled flapping-twisting motion, the wingtip achieved a figure-eight trajectory. Furthermore, by implementing the coupled flapping-swinging motion, an elliptical-like wingtip trajectory was realized. The measurement results are shown in Figure 20, where the arrows indicate the direction of the flapping motion.

A comparison of the wingtip trajectories of the flapping wing and bird wings in Figure 20 reveals a high degree of similarity. This demonstrates that the posture conversion mechanism we designed, through the coordinated operation of multiple motors, can replicate wingtip trajectories similar to those of bird wings, endowing the flapping wing with bird-like flexibility in motion.

### 5.3. Performance Experiment of Attitude Transformation Mechanism

**Figure 21:**
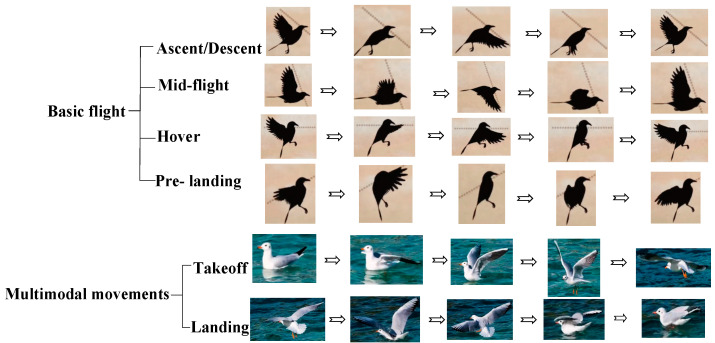

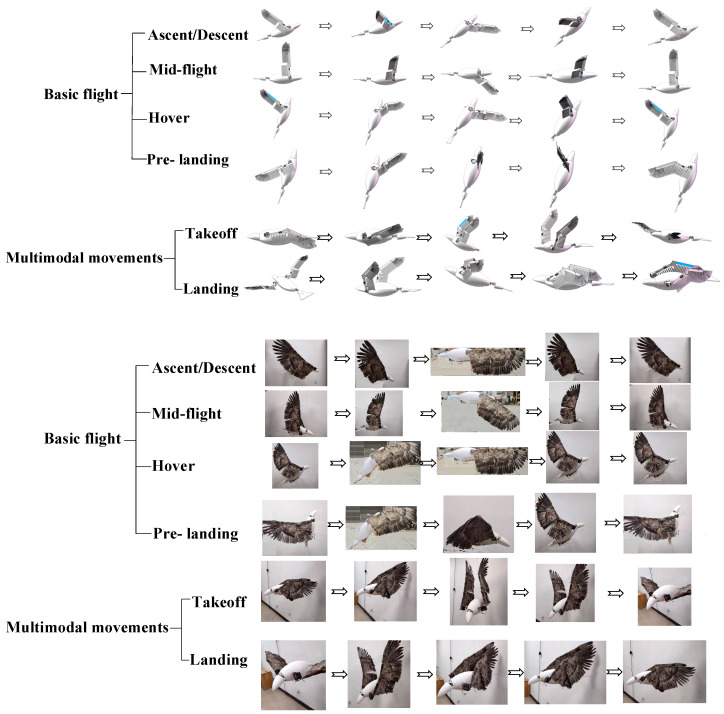
Comparison of basic flight and multimodal movement postures among real birds, the model, and the prototype. The top row displays posture diagrams of different actions in birds, the middle row shows corresponding posture diagrams of the attitude transformation mechanism in the SolidWorks 2022 model, and the bottom row presents the corresponding posture diagrams of the attitude transformation mechanism prototype.

Experiments were conducted on the attitude transformation mechanism to verify its ability to perform basic flight and multimodal movements, including ascent, high-speed flight, hovering, gliding, descent, takeoff, and landing—encompassing a total of 30 primary postures across six motion stages. A comparison of the flight stages and multimodal movements of real birds, the model, and the prototype is shown in Figure 21.

The results indicate that the attitude transformation mechanism effectively replicates the primary postures essential for both basic flight and multimodal movements. This demonstrates that the attitude conversion mechanism designed in this paper enables the robotic bird to exhibit basic flight and multimodal movement abilities similar to those of real birds.

## 6. Conclusions

This paper focuses on the flapping wing module of robotic birds, with the attitude transformation mechanism based on a crank-rocker structure as the research object. The designed mechanism offers high degrees of freedom and ease of installation. When integrated into the robotic bird system, it enables the wings to achieve bird-like movement flexibility. A kinematic model of the attitude transformation mechanism was established, deriving the length constraint conditions for the main linkages necessary to achieve key movements such as flapping, sweeping, and bending. The functionality and accuracy of the kinematic model were validated through experimentation. Experimental results indicate that the designed mechanism satisfies the wing posture requirements during basic flight and multimodal movements, similar to those of birds. The maximum errors in calculating the flapping angle, sweeping angle, and bending angle were 3.4%, 2.7%, and 3.8%, respectively, confirming the accuracy of the kinematic model. The attitude transformation mechanism designed in this paper lays the foundation for advancing robotic birds into the multimodal movement stage. However, this paper has several limitations that should be addressed in future work. First, the experiments were conducted under controlled laboratory conditions, which may not fully replicate real-world flight scenarios, such as varying wind conditions and environmental disturbances. Second, the current design focuses primarily on kinematic performance, while dynamic effects, such as aerodynamic forces and structural vibrations, were not thoroughly investigated. Finally, the scalability of the proposed mechanism to larger or smaller robotic birds remains to be explored. Addressing these limitations in future studies will further enhance the practicality and performance of robotic birds in real-world applications.

## Figures and Tables

**Table 1:** Parameters of the flapping wing.

Parameters	Value
Wingspan (m)	1.21
Wing aera (m^2^)	0.18
Aspect ratio	8
Flapping frequeccy (Hz)	4

**Table 2:** Dimensions of main instruments.

Instruments	Motor	Gearbox	Servo
Type	Maxon DCX 10 L	Maxon GP 42 C	DS1906A
Manufacturer	Maxon	Maxon	GDW
City	Sachseln	Sachseln	Guangzhou
Country	Switzerland	Switzerland	China
Rated speed	9230 rpm	8000 rpm	0.12 s/60°
Maximum torque	1.02 mN∙m	7.5 N∙m	0.196 N∙m
Reduction ratio	None	12:1	None

**Table 3:** Dimensions of main component.

Movement Angle	Component	Value (m)
Flapping angle	*O*_1_ *P*	0.015
	*PQ*	0.04
	*QR*	0.025
	*O*_1_ *R*	0.03
Sweeping angle	*O*_2_ *A*	0.01
	*AB*	0.025
	*BC*	0.023
	*CO*_2_	0.029
Bending angle	*O*_3_ *D*	0.013
	*DE*	0.04
	*EF*	0.015

**Table 4:** Calculation results of movement parameters.

Parameter	Calculation Value/°	Experimental Value/°	Deviation/%
Flapping angle	120	116	3.4
Sweeping angle	105	108	2.7
Bending angle	50	52	3.8

## Data Availability

The data for this paper come from the simulation settings and measurements made during the experiment. Please contact the corresponding author’s email if necessary.
